# Treatment-Refractory New World Cutaneous Leishmaniasis Misdiagnosed as Recurrent Cellulitis in a UK Traveler Returning From the Amazon

**DOI:** 10.7759/cureus.101114

**Published:** 2026-01-08

**Authors:** Amina Khan, Vikram Mahadasa, Bakht Khan

**Affiliations:** 1 Internal Medicine, University Hospital Southampton NHS Foundation Trust, Southampton, GBR; 2 Acute Medicine, University Hospital Southampton NHS Foundation Trust, Southampton, GBR; 3 Acute Medicine, University Hospitals of Derby and Burton NHS Foundation Trust, Derby, GBR

**Keywords:** cutaneous leishmaniasis, meglumine antimoniate, recurrent cellulitis, sporotrichoid, viannia subgenus

## Abstract

Cutaneous leishmaniasis is an uncommon consideration in non-endemic settings, where it may closely resemble bacterial skin infections and result in diagnostic delay. We report the case of a previously healthy young man in the United Kingdom who developed a progressively enlarging ulcer on the dorsum of the hand with associated nodular lymphocutaneous spread following recent travel to the Amazon rainforest. The lesion evolved over several weeks and was repeatedly treated as recurrent cellulitis with multiple courses of antibiotics, without clinical improvement. Histopathological examination and molecular testing ultimately confirmed New World cutaneous leishmaniasis caused by the *Leishmania *(*Viannia*) subgenus. Initial therapy with liposomal amphotericin B was limited by nephrotoxicity and incomplete response, necessitating multidisciplinary review and alternative treatment with meglumine antimoniate, which resulted in complete clinical resolution. This case highlights the diagnostic challenges of cutaneous leishmaniasis in returning travelers and emphasizes the importance of early biopsy, species identification, and specialist involvement when chronic or atypical skin lesions fail to respond to standard antimicrobial therapy.

## Introduction

Leishmaniasis is a protozoal infection transmitted by the bite of infected female phlebotomine sandflies, affecting up to one million individuals worldwide every year. Cutaneous leishmaniasis is globally endemic in parts of the Americas, the Mediterranean basin, the Middle East, Africa, and Central and South Asia [1]. Although the United Kingdom is non-endemic, imported cases are regularly seen in travelers and migrants, mostly acquired in the Mediterranean region and Central and South America. It can present as cutaneous, mucocutaneous, or visceral disease and is endemic in 98 countries, with an estimated 350 million people at risk of infection [2]. Approximately 53 species of *Leishmania* have been identified, 20 of which cause disease in humans, with clinical manifestations ranging from asymptomatic infection to fatal visceral illness.

Cutaneous leishmaniasis lesions can manifest in diverse forms and may mimic a wide range of dermatological conditions, tumors, or cutaneous manifestations of systemic disease, posing a diagnostic dilemma. Although cutaneous leishmaniasis is rarely encountered in the United Kingdom, it should be considered in the differential diagnosis of a new or chronic skin lesion following travel to endemic regions. As of 2025, leishmaniasis remains listed by the World Health Organization as one of the 20 neglected tropical diseases [3]. Increasing global travel, population displacement, and environmental changes have contributed to the emergence of cases in regions where the disease was previously undocumented.

## Case presentation

A man in his early 20s presented to his general practitioner in the United Kingdom with swelling of the left palm and fingers, preceded by a purulent wound on the dorsum of the left hand and painful nodules along the left upper arm. Symptoms had been present for several weeks. Three months prior, he had traveled to Peru, spending five weeks in the Amazon rainforest, with a brief one-day visit to Brazil. During this period, he sustained multiple insect bites and experienced a botfly infestation. The patient was previously healthy, of White ethnicity, with no past medical history including diabetes mellitus, hypertension, or renal disease. He reported no prior history of chronic or recurrent skin lesions. No similar symptoms were identified among household members or close contacts.

Two weeks after returning to the United Kingdom, he noticed a small punctate lesion on the dorsum of his left hand that gradually evolved into a deeper ulcer with rolled edges, measuring approximately 2 cm in diameter. He was initially treated for a presumed infected insect bite with a seven-day course of amoxicillin-clavulanic acid, without improvement. Over subsequent months, he re-presented multiple times for wound care, during which a macerated ulcer with slough, inflamed margins, and areas of granulation tissue was noted. He received additional courses of amoxicillin-clavulanic acid, flucloxacillin, and clarithromycin. Following anaerobic growth on a wound swab, metronidazole was prescribed for a suspected anaerobic infection.

**Figure 1:**
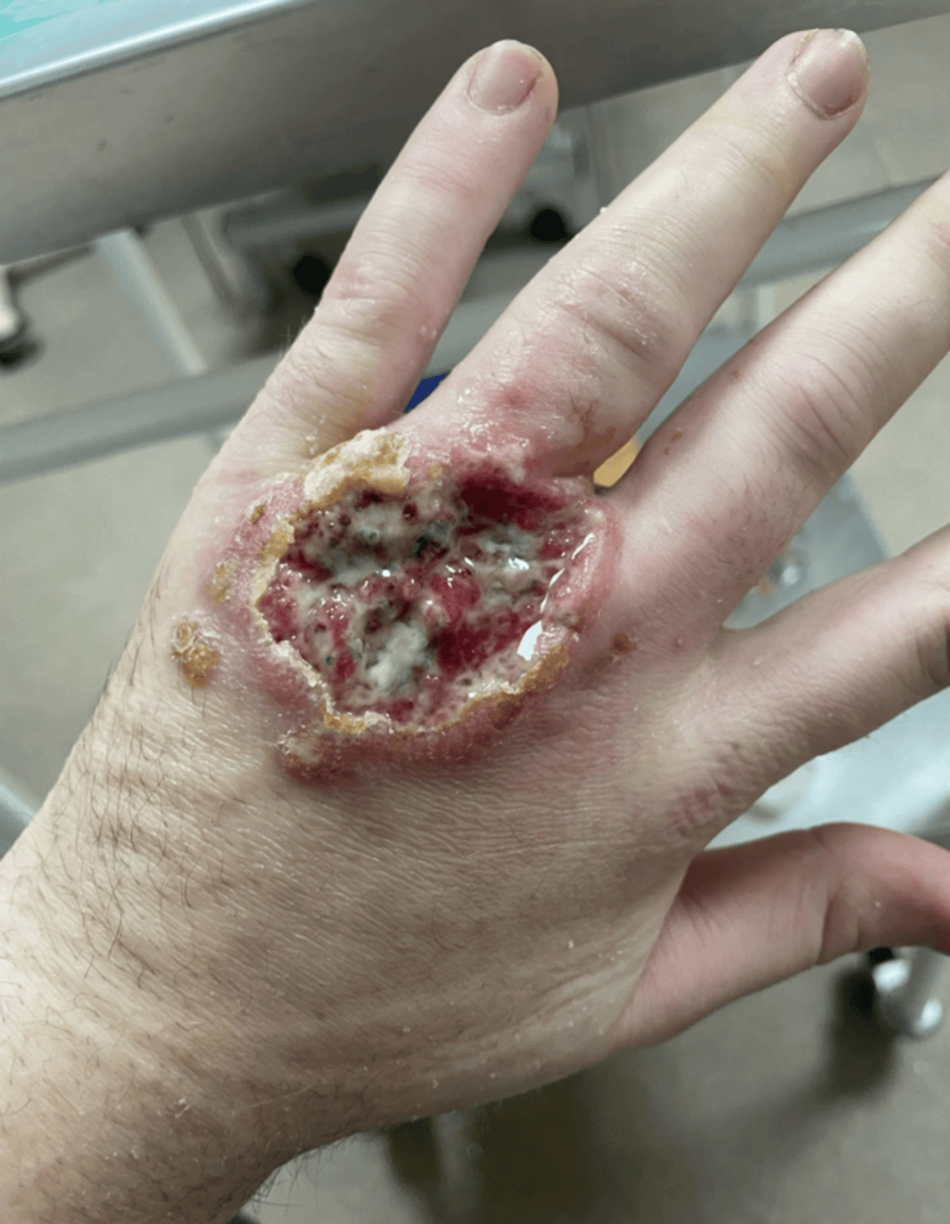
Progressive ulcerative lesion on the dorsum of the left hand three months after onset, following multiple courses of oral antibiotics prescribed for presumed bacterial cellulitis. The lesion measures approximately 4 cm, with a deep ulcer base, raised inflammatory margins, and malodorous serosanguinous discharge

Despite these treatments, the lesion progressed to a deep, painful ulcer with serosanguinous discharge and reduced range of motion of the fingers (Figure 1).

**Figure 2:**
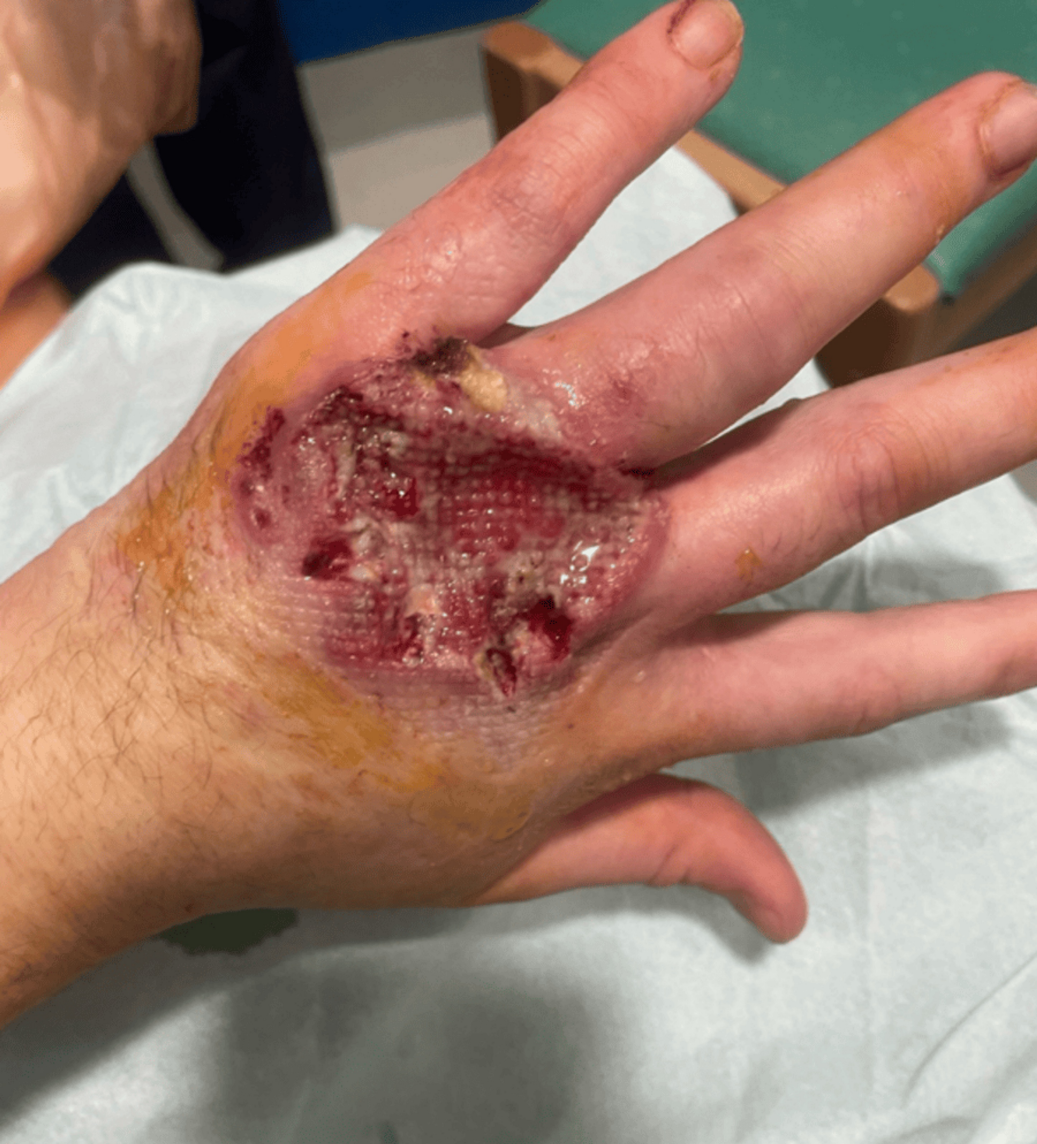
Further deterioration of the lesion with maceration, slough formation, and surrounding erythema with inflamed edges, raising clinical concern for necrotizing soft tissue infection and prompting Plastic Surgery referral

He was referred to the Plastic Surgery outpatient clinic three months after initial presentation due to concern for necrotizing infection, and a biopsy was obtained (Figure 2).

The following day, he presented to the Infectious Diseases Same-Day Emergency Care service due to ongoing diagnostic uncertainty. Clinical examination revealed a 4 cm, heavily exudative, malodorous ulcer on the dorsum of the left hand. Painful nodules tracking along the left arm with palpable left biceps lymphadenopathy were noted, suggestive of sporotrichoid lymphocutaneous spread. Serological testing for HIV, viral hepatitis, strongyloidiasis, and syphilis was negative. Blood glucose levels were within normal limits. Blood tests demonstrated reduced total white blood cell and lymphocyte counts, along with a mild elevation in liver transaminases (Table 1). 

**Table 1:** Summary of laboratory investigations during clinical course Initial investigations demonstrated leukopenia, lymphopenia, and mild transaminitis. Treatment with liposomal amphotericin B was complicated by acute kidney injury. Laboratory parameters normalized following treatment modification and remained within reference ranges during meglumine antimoniate therapy and on follow-up.

Laboratory parameter	At presentation	During amphotericin B therapy	During meglumine antimoniate therapy	On follow-up	Reference range
White blood cell count (×10⁹/L)	3.3	3.7	4.0	4.8	4.0-11.0
Lymphocyte count (×10⁹/L)	0.8	1.5	2	3.8	1.0-4.0
C-reactive protein (mg/L)	3	12	2	1.3	<5
Alanine aminotransferase (U/L)	58	216	56	29	7-55
Serum creatinine (µmol/L)	80	289 (peak)	86	71	64-104

Microscopy of the biopsy specimen revealed characteristic intracellular amastigotes, raising suspicion for leishmaniasis. Polymerase chain reaction (PCR) subsequently detected *Leishmania* DNA of the *Viannia* subgenus, confirming a diagnosis of New World cutaneous leishmaniasis acquired in Peru. Flexible nasal endoscopy performed by the ENT team showed no evidence of mucosal involvement.

The patient was commenced on intravenous liposomal amphotericin B at a dose of 3 mg/kg once daily for five consecutive days, with additional doses planned on days 14 and 21. The patient had no pre-existing renal risk factors. However, after three consecutive infusions, he developed a marked deterioration in renal function consistent with acute kidney injury, necessitating hospital admission and treatment interruption (Table 1). Serum electrolytes, including sodium and potassium, were monitored closely and remained within normal ranges.

**Figure 3:**
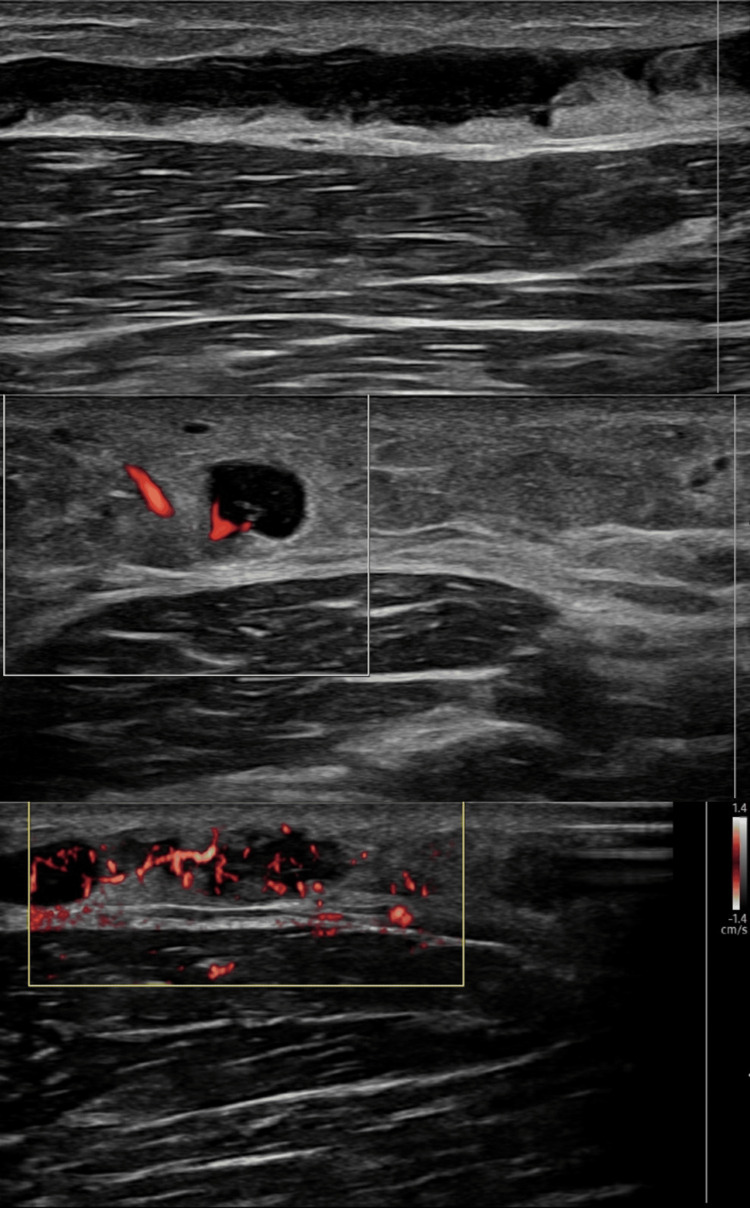
Ultrasound evaluation revealing inflamed superficial veins with intraluminal thrombus and surrounding soft tissue edema, consistent with superficial thrombophlebitis Gray-scale and color Doppler ultrasound images of the left upper antero-medial arm demonstrate dilated, tortuous superficial veins with thickened walls. Color Doppler imaging shows increased hyperaemia within and surrounding the affected vessels. Low-level internal echoes are present within the venous lumen, consistent with intraluminal thrombus. There is associated surrounding subcutaneous soft tissue edema. These sonographic findings are consistent with superficial thrombophlebitis.

The remaining doses were administered twice weekly with close biochemical monitoring and pre-infusion intravenous fluids. Renal function remained stable thereafter, and partial wound improvement was observed, with a reduction in ulcer size. Ultrasonography of the left arm demonstrated superficial thrombophlebitis; deep-vein thrombosis was excluded, and conservative management was instituted (Figure 3). 

**Figure 4:**
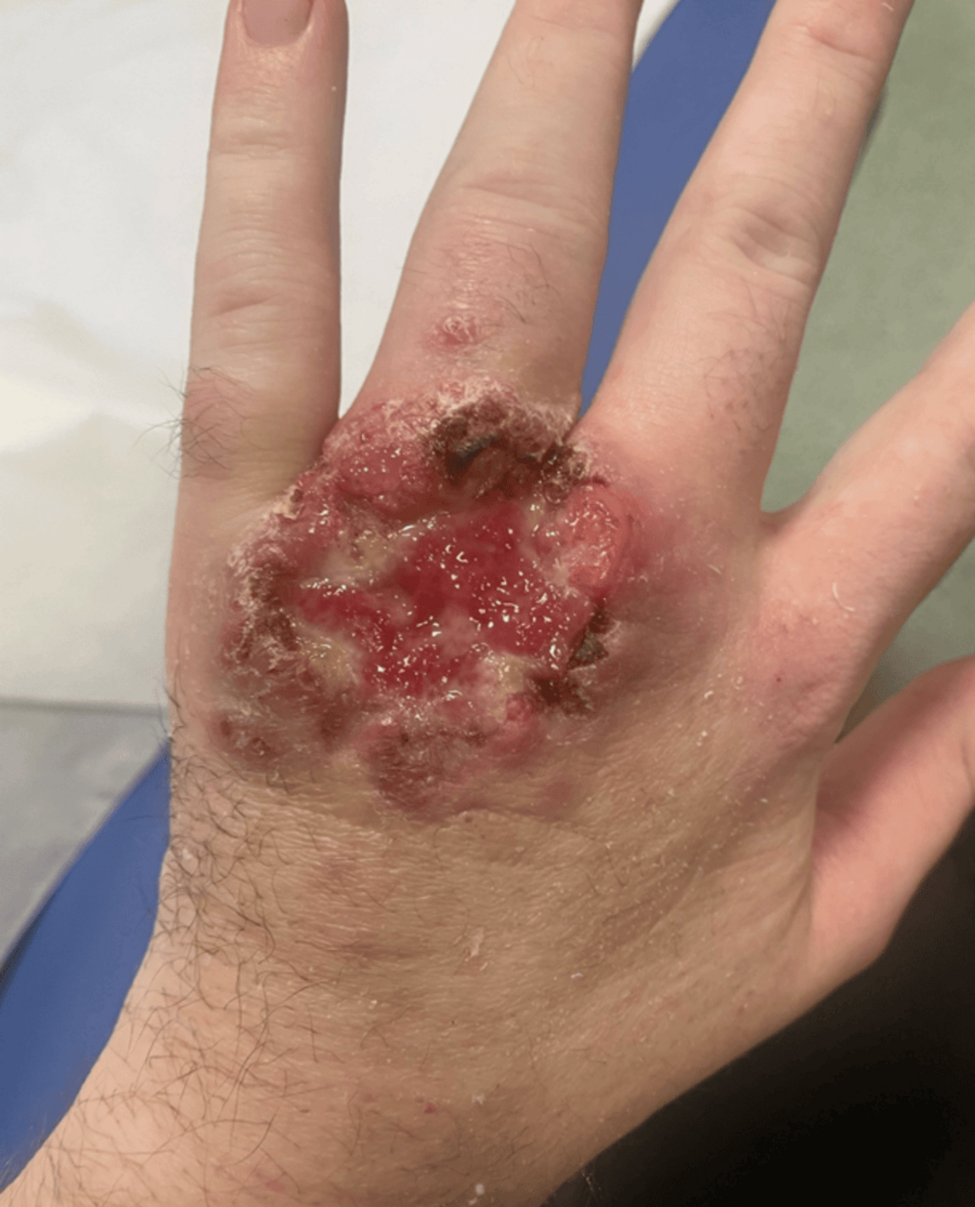
Delayed wound healing with persistent ulceration and granulation tissue, likely reflecting treatment failure secondary to the interrupted dosing of intravenous liposomal amphotericin B due to stage 3 acute kidney injury

At the three-month follow-up, the patient developed two new nodular lesions on the left forearm and demonstrated delayed healing of the primary ulcer (Figure 4).

Repeat biopsy remained PCR-positive for *Viannia* subgenus, indicating incomplete response to treatment. The case was reviewed at the National Leishmaniasis Meeting, where re-treatment with meglumine antimoniate was recommended. Meglumine antimoniate was selected over amphotericin B to avoid potential complications related to renal impairment. Although miltefosine was considered, it was ultimately deemed less favorable due to concerns regarding treatment failure. Given the risk of progression to mucosal disease, prompt re-treatment was considered a clinical priority.

**Figure 5:**
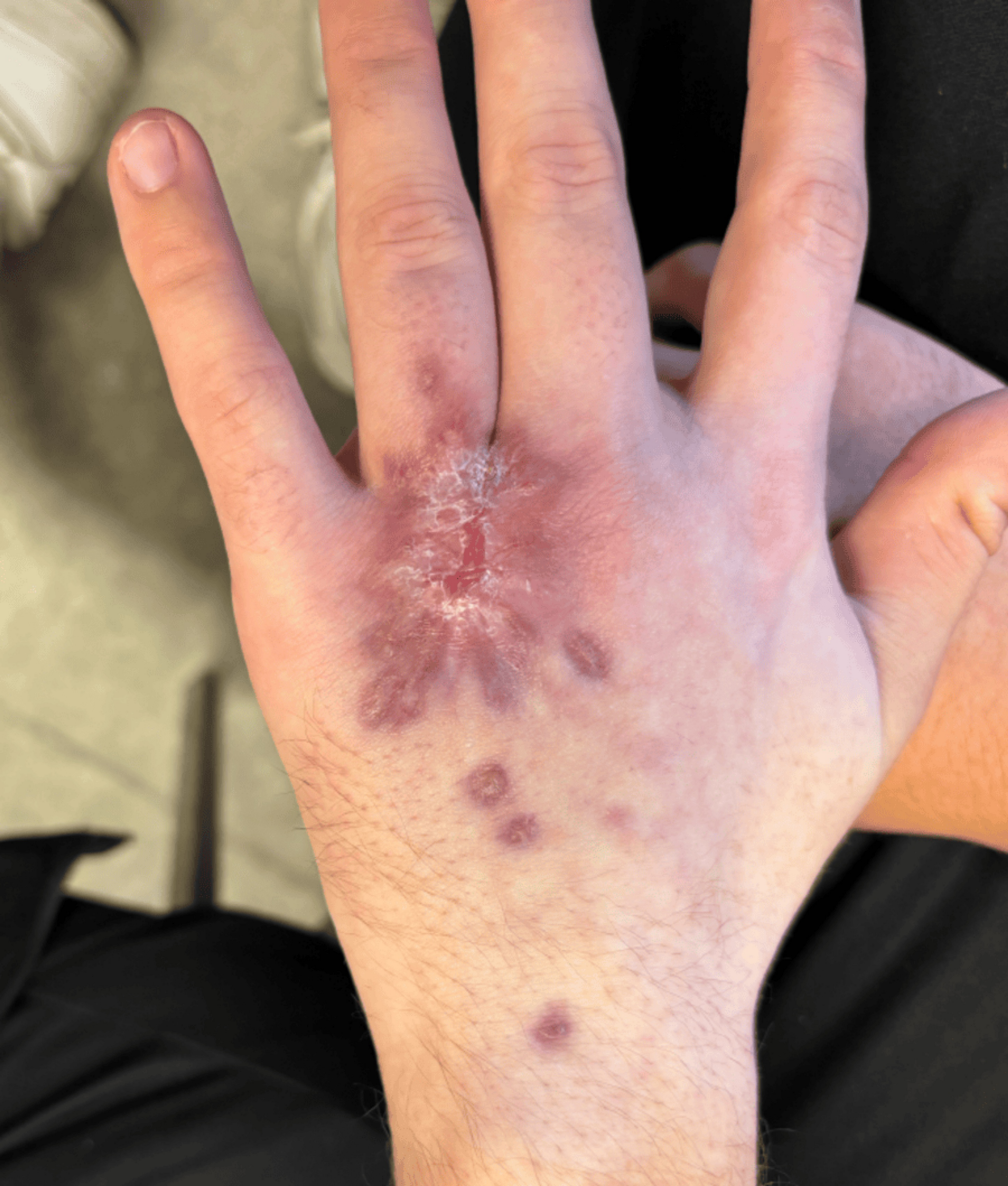
Marked clinical improvement following a 21-day course of intravenous meglumine antimoniate (75 mg/kg/day) administered via peripherally inserted central catheter, demonstrating epithelialization, reduction in lesion size, and resolution of active inflammation

He completed a 21-day course of intravenous meglumine antimoniate at 75 mg/kg/day via a peripherally inserted central catheter. Treatment was well tolerated, with normal biochemical monitoring and electrocardiography throughout. Marked clinical improvement followed, with significant epithelization and healing of the primary ulcer and resolution of sporotrichoid lesions (Figure 5).

A new lesion on the left thigh was biopsied and found to be negative for leishmaniasis on microscopy, culture, and PCR. Repeat ENT examination showed no evidence of mucosal disease. At follow-up, the lesions had healed well with no recurrence, and the patient remained systemically well.

## Discussion

This case highlights the clinical and diagnostic challenges posed by cutaneous leishmaniasis, an ancient disease that continues to perplex clinicians due to its reputation as "the great imitator". Although cutaneous leishmaniasis is often localized, it exhibits a wide spectrum of lesion morphologies, including nodular, ulcerative, eczematoid, hyperkeratotic, sporotrichoid, and plaque-like presentations [4]. The chronicity of these lesions, frequently persisting for weeks to months, predisposes them to secondary bacterial colonization or infection. Positive bacterial cultures from wound exudate can further obscure the underlying diagnosis, leading to repeated and inappropriate antibiotic treatment for presumed cellulitis alone.

The propensity for misdiagnosis is well documented in the literature. Sikorska et al. described a patient with a submandibular ulcer who was treated with antibiotics for nine months following a medical procedure, before a diagnosis of cutaneous leishmaniasis was established [5]. The patient had a history of insect bites sustained during a short jungle hike in Guatemala. Although the lesions ultimately healed following treatment with antimony derivatives, the prolonged diagnostic delay resulted in residual scarring. Such outcomes highlight the importance of increased clinical awareness of leishmaniasis, as delayed diagnosis may lead to disease progression, permanent disfigurement, stigmatization, and significant psychological morbidity. In clinical practice, cutaneous leishmaniasis should be strongly considered in patients presenting with skin lesions that persist for weeks or months, evolve gradually, and fail to respond to multiple courses of antibiotics, particularly in the context of relevant travel exposure. A thorough travel history with potential sandfly exposure remains a cornerstone of diagnosis.

The *Viannia* subgenus includes *Leishmania braziliensis* and *Leishmania guyanensis*, which are endemic to the Amazon basin, including Peru, Brazil, the Guianas, and Venezuela, with *Leishmania guyanensis* representing one of the most frequent causes of cutaneous leishmaniasis in this region [6]. Accurate identification of the etiologic species is essential, as infection with certain species carries a risk of visceral or mucosal involvement and treatment response may vary significantly even among isolates acquired from different endemic areas. Although diagnostic modalities such as culture, histopathology, and PCR are readily available, the greatest diagnostic challenge remains the clinician's ability to suspect leishmaniasis early in the disease course.

Transmission of *Leishmania *species primarily occurs through the bite of infected phlebotomine sandflies; however, rare cases of human-to-human transmission via infected needles, blood transfusion, or congenital routes have been reported. In the New World, reservoir hosts include mammals such as rodents and canines, with higher prevalence traditionally observed in rural areas with minimal deforestation. However, climate change, environmental disruption, and increasing rates of immunosuppression have enabled vector adaptation to suburban and urban environments [6]. Close proximity between humans, pets, and livestock may further increase transmission pressure by maintaining untreated reservoirs. Ultimately, the clinical manifestations of leishmaniasis reflect a complex interplay between parasite virulence factors and the host immune response. Mahdavi et al. reported a rare case of disseminated cutaneous leishmaniasis with recurrence four years after the initial lesion in a patient receiving immunosuppressive therapy [7]. Immunosuppression is a well-recognized risk factor for severe, atypical, and disfiguring presentations of leishmaniasis, including those resulting in functional impairment.

This case demonstrates a classic presentation of sporotrichoid lymphocutaneous dissemination, also known as nodular lymphangitis. Leishmaniasis is among the conditions that follow this characteristic lymphatic spread, historically associated with *Sporothrix schenckii* infection [8]. Following inoculation, a small nodule typically develops within 2-24 weeks and may progress to a shallow, well-demarcated ulcer. Nodular lymphangitis is particularly associated with the *Viannia *subgenus and reflects an inflammatory response adjacent to lymphatic vessels, which may predispose to thrombotic complications, as observed in this case. Although thrombotic phenomena, including disseminated intravascular coagulation, are more commonly described in visceral leishmaniasis [9], the presence of superficial thrombophlebitis in cutaneous disease underscores the need for heightened vigilance. This finding suggests a low threshold for evaluating thrombotic complications and considering thromboprophylaxis in patients with additional risk factors.

This case also highlights the considerable challenges associated with the treatment of leishmaniasis. Available therapeutic options are limited by adverse effects, emerging drug resistance, and the requirement for prolonged parenteral administration [10]. Pentavalent antimonials were historically considered first-line therapy; however, their use in New World cutaneous leishmaniasis has declined due to increasing resistance and treatment failure. Commonly reported adverse effects include myalgia, arthralgia, hepatotoxicity, nephrotoxicity, QT interval prolongation, ST-segment depression, and sinus bradycardia [6]. As a result, liposomal amphotericin B is often favored as an alternative treatment for New World cutaneous leishmaniasis, particularly in resistant cases.

Despite its efficacy, amphotericin B is associated with significant toxicity, most notably nephrotoxicity, and requires careful monitoring and inpatient administration. In this case, although pre-infusion intravenous hydration improved tolerability, treatment interruption and dose spacing due to renal injury likely contributed to therapeutic failure. Miltefosine was considered as an alternative option due to its oral administration and favorable efficacy; however, concerns regarding teratogenicity and emerging resistance limited its use. Ultimately, after weighing risks and benefits, the multidisciplinary leishmaniasis team elected to proceed with a full course of the pentavalent antimonial meglumine antimoniate. Sequential therapy resulted in complete clinical resolution, illustrating the variable therapeutic response of *Leishmania *species and reinforcing the importance of individualized, multidisciplinary decision-making in complex cases.

## Conclusions

Cutaneous leishmaniasis should be considered in returning travelers who present with chronic, non-healing skin lesions, particularly when there is poor response to repeated antibiotic therapy or evidence of sporotrichoid lymphocutaneous spread. Early biopsy and species identification are essential, as clinical behavior, treatment response, and risk of complications vary by *Leishmania *subgenus. Management can be challenging due to drug toxicity, treatment failure, and the need for prolonged parenteral therapy, emphasizing the importance of multidisciplinary involvement. Increasing global travel, environmental change, and expanding vector distribution make early recognition critical to prevent diagnostic delay, disease progression, and long-term morbidity.
